# Risk factors associated with SGLT2 inhibitor discontinuation in diabetic patients with heart failure

**DOI:** 10.1371/journal.pone.0314305

**Published:** 2024-11-25

**Authors:** Minkwan Kim, Seok-Jae Heo, Moon-Hyun Kim, Je-Wook Park, SungA Bae, Ji Woong Roh, Oh-Hyun Lee, Yongcheol Kim, Eui Im, In Hyun Jung, Deok-Kyu Cho

**Affiliations:** 1 Division of Cardiology, Department of Internal Medicine, Yongin Severance Hospital, Yonsei University College of Medicine, Yongin, Gyeonggi-do, Republic of Korea; 2 Division of Biostatistics, Department of Biomedical Systems Informatics, Yonsei University College of Medicine, Seoul, Republic of Korea; Army Medical University, CHINA

## Abstract

Sodium-glucose cotransporter 2 inhibitors (SGLT2i), have shown benefits in patient with heart failure (HF), however, adherence remains a significant issue: with only 60% of patients continuing usage beyond a year. This study aims to identify patients at risk of discontinuing SGLT2i and promote its judicious use to reduce hospitalizations and improve cardiovascular outcomes. Using the Korean National Health Insurance Service database, patients diagnosed with HF and diabetes mellitus (n = 1,665,565) between 2013 and 2018 were identified. Among them, 55,694 participants prescribed SGLT2i were enrolled. The primary endpoint included 1) all-cause mortality and 2) SGLT2i-related hospitalization, encompassing incidents such as ketoacidosis, acute kidney injury, urinary tract infections, fall-related fractures, and other unplanned hospitalizations. During the follow-up period (median: 2.3 years; range: 1.2–3.6 years), 8,463 participants reached the primary endpoint (25.5 for all-cause death and 39.4 for SGLT2i-related hospitalizations per 1,000 person-years). Independent risk factors for the primary endpoint in multivariate Cox regression and propensity-score matching analyses included age of ≥ 70 years, body mass index (BMI) <18.5 kg/m^2^, body weight <60 kg, anemia, chronic kidney disease, and the use of diuretics. Age (hazard ratio [HR] 1.45, 95% confidence interval [CI]: 1.36–1.54), BMI (HR 1.78, 95% CI: 1.29–2.45), body weight (HR 1.17, 95% CI: 1.09–1.26) and the use of furosemide (HR 1.45, 95% CI: 1.22–1.74) (all *p*<0.001) were consistent independent risk factors in the propensity score-matched cohort. Having three or more risk factors was associated with an adjusted HR that was 3.04 times higher than cases with no risk factor (95% CI: 2.83–3.28, *p*<0.001). Old age, low weight or BMI, and the use of diuretics are risk factors that hinder the continuous use of SGLT2i in diabetic patients with HF. Close monitoring for side effects is essential when prescribing SGLT2i, particularly for those with multiple risk factors.

## Introduction

Sodium-glucose cotransporter 2 inhibitors (SGLT2i), originally developed as antidiabetic drugs, have gained widespread usage owing to their proven ability to ameliorate disease conditions, delay disease progression, and confer positive prognostic effects in patients with heart failure (HF) and chronic kidney disease [1–4]. The recent rise in life expectancy has led to a corresponding increase in the prevalence of HF with preserved ejection fraction (HFpEF). Notably, studies have shown that SGLT2i can reduce cardiovascular composite events in HFpEF, suggesting an anticipated increase in their prescription [5, 6]. Moreover, similar results have been observed in patients without diabetes, suggesting that the beneficial cardiovascular outcomes of SGLT2i may be attributed to mechanisms beyond their glucose-lowering effects. Ongoing studies are actively investigating these mechanisms [1, 5–7].

Furthermore, studies assessing the effects or safety of SGLT2i have not revealed a significantly increased risk of adverse effects when compared to placebos [5, 6, 8–12]. However, in clinical practice, challenges arise as numerous patients discontinue SGLT2i use due to side effects, while others find it arduous to adhere to the prescribed regimen. In a recent study involving 120,000 participants, only 60% continued to take SGLT2i consistently for longer than a year [13]. Current guidelines strongly recommend the use of SGLT2i in diabetic patients with HF [14, 15].

However, given that the baseline characteristics of participants enrolled in the foundational clinical trials may differ from those of patients in real-world clinical settings who are candidates for SGLT2i treatment, it is expected that adherence rates may be lower, as indicated by the aforementioned meta-analysis results [13]. Therefore, we aimed to identify which patients have a higher propensity to discontinue SGLT2i or for whom SGLT2i should be prescribed judiciously. With this approach, we aim to prevent rising medical costs due to hospitalizations resulting from drug-related side effects and promote a reduction in cardiovascular events through appropriate HF treatment.

## Methods

### Data source and study population

**Fig 1:**
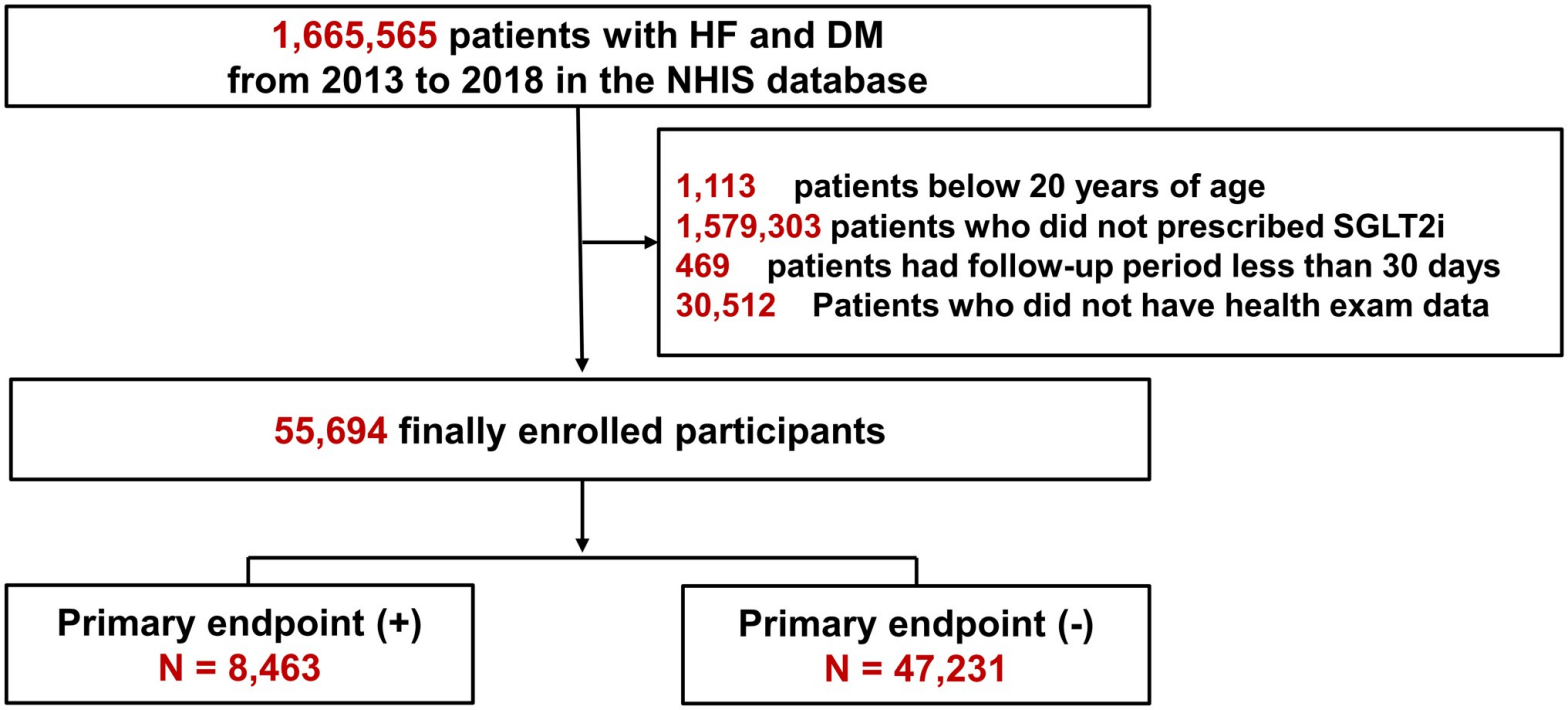
Study flow. HF, heart failure; DM, diabetes mellitus.

We conducted a nationwide cohort study in the Republic of Korea, utilizing the claims database of the National Health Insurance Service (NHIS). The NHIS, a compulsory health insurance scheme, has been under the Korean government’s supervision since 1989, providing medical services to an impressive 97% of the population [16]. This database, meticulously maintained by the NHIS, contains detailed information for each registered individual, encompassing demographic profiles, health-related behaviors, medical diagnoses, prescribed medications, surgical or procedural history, and records of healthcare usage such as hospital admissions [16]. The reliability of this database as a robust data source has been validated through its extensive use in published research papers [17, 18]. From the NHIS database, we identified patients diagnosed with both HF and diabetes mellitus between January 1, 2013 and December 31, 2018, totaling 1,665,565 patients. After excluding 1,113 patients below 20 years of age, 1,579,303 patients who were not prescribed SGLT2i, and 469 patients with a follow-up period of < 30 days, a total of 55,694 participants were enrolled in this study (Fig 1). This research adhered to the principles outlined in the 2013 Declaration of Helsinki. Our hospital’s Institutional Review Board granted approval for the study, and given its retrospective design, the requirement for informed consent was waived. The data were accessed for research purposes from May 23, 2023, to October 31, 2023. The information we accessed was de-identified data with personal details of the participants removed.

### Covariates

This study incorporated methods outlined in previously published studies [19, 20]. Data on age and sex were extracted using resident identification numbers. Income level was categorized into quartiles. Comorbidities, including hypertension, dyslipidemia, revascularization-requiring ischemic heart disease, atrial fibrillation, ischemic/bleeding stroke, and chronic kidney disease, were operationally defined, as described in S1 Table in S1 File. The Charlson Comorbidity Index was calculated following previously described methods [21, 22]. Prescribed medications, such as renin-angiotensin-aldosterone system blockers, which include angiotensin receptor-neprilysin inhibitors, beta-blockers, calcium channel blockers, statins, spironolactone, and antiplatelet agents, were identified from prescription records in the database. Anemia was defined as hemoglobin levels < 12.0 g/dL in women and < 13.0 g/dL in men, in accordance with the World Health Organization guidelines [23]. Estimated glomerular filtration rate (eGFR) was determined using the formula provided by the Chronic Kidney Disease Epidemiology Collaboration [24].

### Study outcome

The primary endpoint comprised 1) death from any cause and 2) SGLT2i-related admissions. The latter encompassed incidents of diabetic ketoacidosis (DKA), acute kidney injury (AKI), urinary tract infections (including acute pyelonephritis), falls or fall-related fractures, and other unplanned hospitalizations due to unidentified causes. The category of unplanned hospitalizations serves as an operational definition to identify patients who discontinue SGLT2i due to adverse events or intolerance and includes cases where patients experience serious adverse events requiring hospitalization and discontinue SGLT2i therapy for more than three months both after discharge and during ongoing outpatient care. Cases in which patients with no recent hospitalization where patients reported discomfort and discontinued SGLT2i in the outpatient clinic were not included under this definition. To assess the risk of adverse effects and mortality associated with SGLT2i usage in patients with HF, we determined the index date based on the earlier of two conditions: 1) For patients who were taking SGLT2i prior to their initial diagnosis of HF, the index date was set as the date of their first HF diagnosis; 2) For those who began taking SGLT2i after their initial diagnosis of HF, the index date was the commencement date of SGLT2i usage. For patients who experienced both SGLT2i-induced admissions and all-cause death events, the first event, i.e. SGLT2i-induced admission, was included in the primary endpoint analysis.

For the sensitivity analysis, we examined the analysis in patients enrolled in the rare intractable disease program for heart failure (code V127) with ICD-10 disease codes I42.0 to I42.2, using the same analysis format as applied to the total population. These patients were categorized into three subgroups: (1) patients with left ventricular ejection fraction < 45% and pulmonary congestion evident on chest radiographs (I42.0), (2) patients diagnosed with dilated cardiomyopathy (I42.0), and (3) patients diagnosed with hypertrophic cardiomyopathy (I42.1, I42.2). As part of the NHIS’s enhanced assistance initiative, these patients received extended privileges and support. The diagnoses were validated by the attending physician and the Health Insurance Review and Assessment Service (HIRA), ensuring high accuracy [25].

### Statistical analysis

Descriptive statistics were presented as means ± standard deviations for continuous measures, and numbers (proportions) for categorical measures. The independent Student’s *t*-test was utilized for continuous variables, while the chi-squared test or Fisher’s exact test was employed for categorical measures, as appropriate, to compare between groups. The incidence rates of primary endpoint were calculated by dividing the number of detected cases by the follow-up duration and were presented as a value per 1,000 person-years. Kaplan-Meier curves were used to present the cumulative incidence of the primary and secondary endpoints, with a log-rank test for statistical analysis. Multivariable Cox proportional-hazards analysis was used to identify the independent risk factors for the primary endpoint, which were expressed as HRs and corresponding 95% confidence intervals (CIs). For propensity score-matching analysis, we balance the differences in covariates related to SGLT2i usage, except for one variable that independently influenced SGLT2i cessation. We employed nearest-neighbor matching with a caliper size 0.1 standard deviation of logit of the propensity scores. All statistical analyses were conducted using SAS version 9.3 (SAS Institute) and R software (version 3.6.3; R Foundation for Statistical Computing, Vienna, Austria). A two-sided *p*-value of < 0.05 was considered statistically significant.

## Results

### Baseline characteristics of the study participants

A total of 55,694 participants were included in this study, with an average age of 63.2 ± 10.8 years, and 43.0% were female. The participants’ baseline clinical characteristics are presented in Table 1. Generally, participants who reached the primary endpoint were older (67.6 ± 10.7 vs. 62.4 ± 10.6, *p* < 0.001) and had lower incomes. Moreover, they were more likely to have hypertension, ischemic heart disease, atrial fibrillation, a history of cerebrovascular incidents, a higher Charlson Comorbidity Index (2.31 ± 1.98 vs. 1.95 ± 1.89, *p* < 0.001), higher serum fasting glucose levels, and a higher usage of angiotensin receptor-neprilysin inhibitors, spironolactone, and loop diuretics. However, participants who reached the primary endpoint were less likely to have obesity and lower body weight, estimated glomerular filtration rate, and hemoglobin (Table 1).

**Table 1:** Baseline characteristics of SGLT2i-treated diabetic patients with heart failure, stratified by the primary endpoint occurrence.

	Primary endpoint (+) (N = 8,463)	Primary endpoint (-) (N = 47,231)	*p*-value
Age, years	67.6 ± 10.7	62.4 ± 10.6	< 0.001
Age ≥ 70-year-old	5312 (62.8)	20461 (43.3)	< 0.001
Female sex	3696 (43.7)	20320 (43.0)	0.271
Income, low 20%	2367 (28.0)	11481 (24.3)	< 0.001
Body mass index, kg/m^2^	26.0 ± 4.3	27.0 ± 4.2	< 0.001
< 18.5	3538 (41.8)	15077 (31.9)	< 0.001
18.5–24.9	4766 (56.3)	31905 (67.6)	
≥ 25.0	159 (1.9)	249 (0.5)	
Body weight, kg	67.3 ± 13.4	71.6 ± 14.1	< 0.001
< 60	2422 (28.6)	9040 (19.1)	< 0.001
*Underlying disease*, *n (%)*			
Hypertension	6754 (79.8)	36612 (77.5)	< 0.001
Dyslipidemia	8018 (94.7)	45364 (96.0)	< 0.001
Ischemic heart disease	5279 (62.4)	25718 (54.5)	< 0.001
Atrial fibrillation	1435 (17.0)	5755 (12.2)	< 0.001
Previous stroke	1291 (15.3)	4198 (8.9)	< 0.001
Chronic kidney disease	3955 (46.7)	16180 (34.3)	< 0.001
*Medication*, *n (%)*			
RAS blocker	6077 (71.8)	34458 (73.0)	0.030
Beta-blocker	4605 (54.4)	25831 (54.7)	0.645
ARNI	199 (2.4)	915 (1.9)	0.014
Spironolactone	1623 (19.2)	5363 (11.4)	< 0.001
Loop diuretics	2804 (32.6)	8579 (18.3)	<0.001
Statin	2756 (32.6)	8689 (18.4)	< 0.001
*Laboratory findings*, *n (%)*			
Hemoglobin, g/dL	13.5 ± 1.9	14.2 ± 1.7	< 0.001
< 13 (male) or < 12 (female)	2197 (26.0)	5874 (12.4)	< 0.001
Fasting glucose, mg/dL	149.1 ± 62.2	144.4 ± 49.2	< 0.001
≥ 126	4971 (58.7)	28142 (59.6)	0.148
Creatinine, mg/dL	1.04 ± 1.18	0.94 ± 0.73	<0.001
Estimated GFR, mL/min/1.73m^2^	75.9 ± 26.8	83.0 ± 26.0	< 0.001
Charlson comorbidity index	7.2 ± 2.9	5.8 ± 2.4	< 0.001

RAS, renin-angiotensin system; ARNI, angiotensin receptor-neprilysin inhibitor; GFR, glomerular filtration rate

### Study endpoints in the original cohort

**Fig 2:**
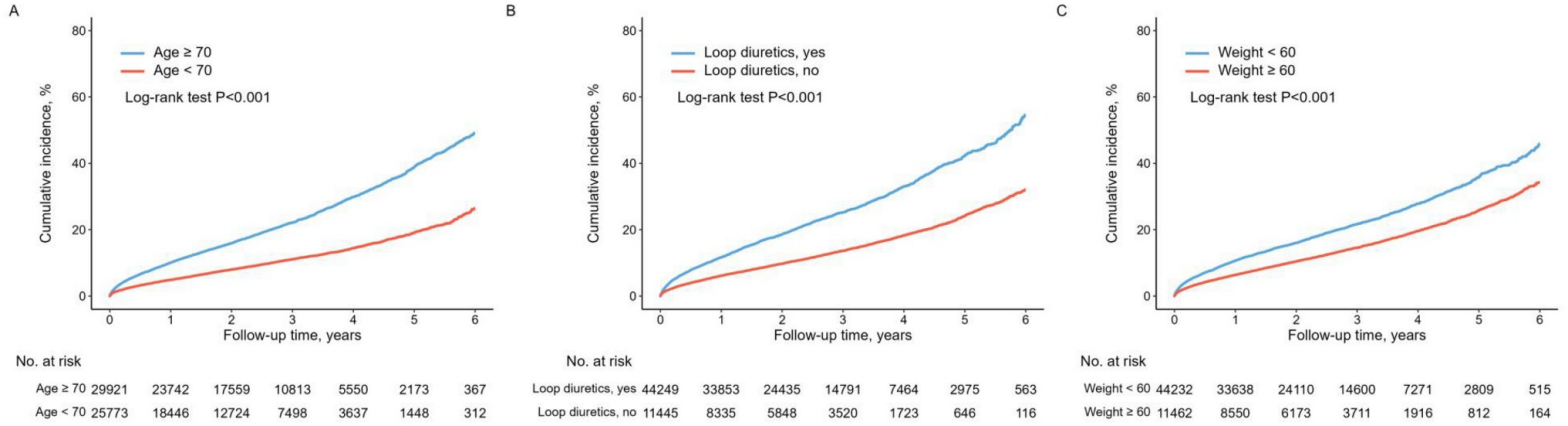
Kaplan–Meier curve of the cumulative incidence of the primary endpoint according to (A) age of 70 or older, (B) use of loop diuretics, (C) low body weight below 60 kg.

The median follow-up duration was 2.3 (IQR: 1.2–3.6) years. During the follow-up period, the primary endpoint was reached in 8,436 (15.2%) patients or 65 patient-years. All-cause death occurred in 3,327 (6.0%) patients, and SGLT2i-induced admissions were observed in 5,143 (9.2%) patients. Admissions due to DKA accounted for 27 (0.05%) cases, AKI for 425 (0.8%), and falls and fractures for 302 (0.5%). Moreover, 4,125 patients (7.4%) discontinued SGLT2i after admission for unknown reasons (Table 2). To determine the risk factors for the primary endpoint, a multivariable Cox analysis model was employed (Table 3). Age ≥ 70 years (adjusted HR: 1.66, 95% CI: 1.59–1.75, *p* < 0.001), BMI < 18.5 kg/m^2^ (adjusted HR: 1.82, 95% CI: 1.54–2.13, *p* < 0.001) body weight < 60 kg (adjusted HR: 1.25, 95% CI: 1.18–1.31, *p* < 0.001), anemia (adjusted HR: 1.41, 95% CI: 1.34–1.48), eGFR < 60mL/min/1.73m^2^ (adjusted HR: 1.21, 95% CI: 1.15–1.27, *p* < 0.001), use of loop diuretics (adjusted HR: 1.38, 95% CI: 1.30–1.46, *p* < 0.001) and spironolactone (adjusted HR: 1.20, 95% CI: 1.12–1.28, *p* < 0.001) were identified as independent predictors of the primary endpoint. The fasting glucose level did not exhibit statistical significance as an independent predictor for adverse reactions leading to SGLT2i discontinuation. According to the Kaplan-Meier survival curves, participants aged ≥ 70, those with body weight < 60 kg, and users of loop diuretics had a higher incidence of the primary endpoint than those without these characteristics (all log-rank *p* < 0.001) (Fig 2).

**Table 2:** Clinical outcome in the original cohort of diabetic patients with heart failure.

	Events (%)	Incidence rate (per 1,000)
**Primary endpoint**	8463 (15.2)	25.36
All-cause death	3327 (6.0)	22.83
SGLT2i-induced admission	5143 (9.2)	34.15
Ketoacidosis	27 (0.05)	5.78
Acute kidney injury	425 (0.8)	5.52
Urinary tract infection, including pyelonephritis	286 (0.5)	6.67
Slipdown and/or fracture	302 (0.5)	21.33
Other unplanned admission	4125 (7.4)	18.69

**Table 3:** Cox regression analysis of original and propensity score-matched cohorts of SGLT2i-treated diabetic patients with HF.

Variables		Univariable Cox		Multivariable Cox*		Propensity score-matched cohort*	
		HR (95% CI)	*p*	HR (95% CI)	*p*	HR (95% CI)	*p*-value
Age ≥ 70 years	Primary endpoint	2.19 (2.10–2.29)	< 0.001	1.66 (1.59–1.75)	< 0.001	1.45 (1.36–1.54)	< 0.001
	All-cause death	3.94 (3.65–4.27)	< 0.001	2.764 (2.54–3.01)	< 0.001	2.74 (2.32–3.23)	< 0.001
	SGLT2i-induced admission	1.57 (1.48–1.66)	< 0.001	1.25 (1.18–1.33)	< 0.001	1.27 (1.19–1.36)	< 0.001
BMI < 18.5 kg/m^2^	Primary endpoint	3.01 (2.57–3.52)	< 0.001	1.82 (1.54–2.13)	< 0.001	1.78 (1.29–2.45)	< 0.001
	All-cause death	4.97 (4.05–6.08)	< 0.001	2.24 (1.81–2.77)	< 0.001	3.14 (1.72–5.74)	< 0.001
	SGLT2i-induced admission	1.88 (1.46–2.41)	< 0.001	1.41 (1.10–1.82)	0.008	1.30 (0.89–1.90)	0.179
Body weight < 60kg	Primary endpoint	1.54 (1.47–1.61)	< 0.001	1.25 (1.18–1.31)	< 0.001	1.17 (1.09–1.26)	< 0.001
	All-cause death	1.90 (1.77–2.04)	< 0.001	1.55 (1.43–1.68)	< 0.001	1.73 (1.46–2.04)	< 0.001
	SGLT2i-induced admission	1.33 (1.25–1.41)	< 0.001	1.08 (1.01–1.16)	0.022	1.07 (0.99–1.16)	0.086
Fasting glucose ≥ 126mg/dL	Primary endpoint	0.93 (0.90–0.98)	0.002	1.04 (1.00–1.09)	0.084	1.00 (0.94–1.05)	0.865
	All-cause death	0.98 (0.91–1.05)	0.541	1.15 (1.07–1.23)	< 0.001	1.07 (0.94–1.21)	0.314
	SGLT2i-induced admission	0.91 (0.86–0.96)	< 0.001	0.98 (0.93–1.04)	0.473	0.98 (0.92–1.04)	0.489
Hemoglobin < 13 (male) or < 12 (female) g/dL	Primary endpoint	2.09 (1.99–2.19)	< 0.001	1.41 (1.34–1.48)	< 0.001	1.32 (1.22–1.43)	< 0.001
		2.81 (2.61–3.02)	< 0.001	1.616 (1.50–1.75)	< 0.001	1.78 (1.49–2.11)	< 0.001
	All-cause death	1.67 (1.56–1.79)	< 0.001	1.25 (1.16–1.34)	< 0.001	1.21 (1.10–1.32)	< 0.001
	SGLT2i-induced admission	1.69 (1.62–1.77)	< 0.001	1.21 (1.15–1.27)	< 0.001	1.17 (1.09–1.25)	< 0.001
Estimated GFR < 60mL/min/1.73m^2^	Primary endpoint	2.35 (2.19–2.51)	< 0.001	1.28 (1.19–1.39)	< 0.001	1.29 (1.12–1.49)	< 0.001
	All-cause death	1.37 (1.29–1.443)	< 0.001	1.17 (1.10–1.25)	< 0.001	1.14 (1.06–1.22)	< 0.001
	SGLT2i-induced admission	1.98 (1.90–2.08)	< 0.001	1.38 (1.30–1.46)	< 0.001	1.36 (1.25–1.47)	< 0.001
Use of loop diuretics	Primary endpoint	2.53 (2.36–2.71)	< 0.001	1.57 (1.45–1.71)	< 0.001	1.45 (1.22–1.74)	< 0.001
	All-cause death	1.68 (1.59–1.79)	< 0.001	1.26 (1.17–1.35)	< 0.001	1.33 (1.21–1.46)	< 0.001
	SGLT2i-induced admission	1.81 (1.72–1.91)	< 0.001	1.20 (1.12–1.28)	< 0.001	1.20 (1.10–1.31)	< 0.001
Use of spironolactone	Primary endpoint	2.12 (1.95–2.30)	< 0.001	1.19 (1.08–1.32)	< 0.001	1.24 (1.03–1.50)	0.027
	All-cause death	1.63 (1.52–1.75)	< 0.001	1.22 (1.12–1.33)	< 0.001	1.20 (1.09–1.32)	< 0.001
	SGLT2i-induced admission	2.19 (2.10–2.29)	< 0.001	1.66 (1.59–1.75)	< 0.001	1.45 (1.36–1.54)	< 0.001

* Multivariate Cox and propensity score-matching model were adjusted for age, sex, income, BMI, Charlson Comorbidity Index, hypertension, dyslipidemia, ischemic heart disease, atrial fibrillation, stroke, use of renin-angiotensin system blockade, beta-blockers, calcium channel blocker, antiplatelet agent, angiotensin receptor-neprilysin inhibitor, MRA, statins, loop diuretics, level of hemoglobin, serum glucose, creatinine, estimated GFR. In each statistical test, independent variables were excluded from the adjustment. HR, hazard ratio

**Fig 3:**
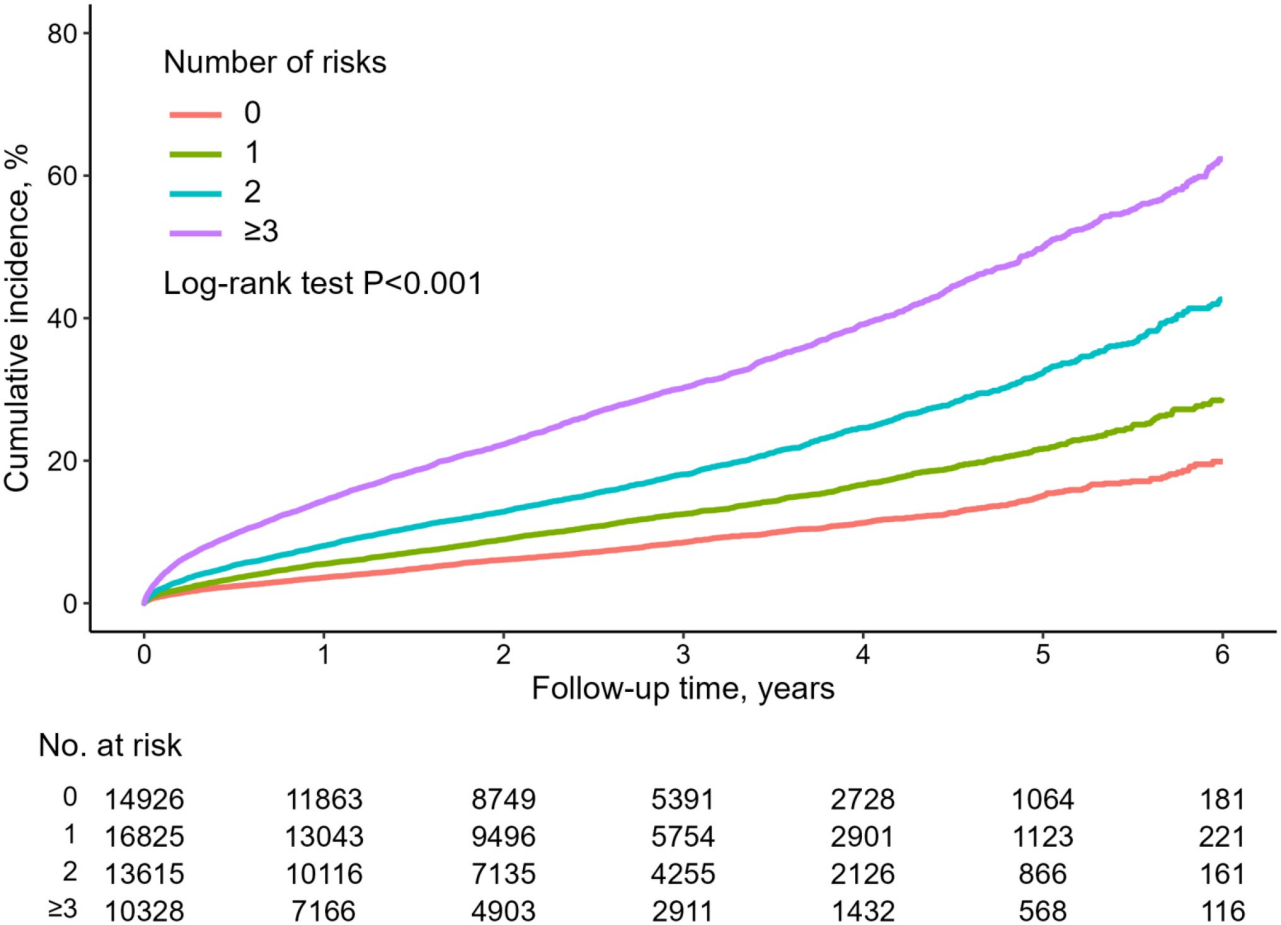
Kaplan–Meier curve of the cumulative incidence of the primary endpoint according to the number of risk factors.

The risk of the composite primary endpoint, based on the number of significant risk factors previously mentioned, is shown in Table 4. Having three or more risk factors was associated with an adjusted HR that was 3.04 times higher compared to cases with only one risk factor (95% CI: 2.83–3.28, *p* < 0.001). Furthermore, as the number of significant risk factors increased, there was a tendency for more primary endpoints to occur (log-rank *p* < 0.001) (Fig 3).

**Table 4:** Cox regression analysis in the original cohort estimating primary outcomes according to risk factor numbers.

The number of risk factors	Outcomes	Unadjusted		Adjusted*	
		HR (95% CI)	*p*-value	HR (95% CI)	*p*-value
0 (n = 14926)	Primary endpoint	Reference		Reference	
	All-cause death				
	SGLT2i-induced admission				
1 (n = 16825)	Primary endpoint	1.52 (1.41–1.63)	< 0.001	1.38 (1.29–1.49)	< 0.001
	All-cause death	2.25 (1.94–2.62)	< 0.001	2.03 (1.75–2.37)	< 0.001
	SGLT2i-induced admission	1.33 (1.22–1.44)	< 0.001	1.21 (1.11–1.32)	< 0.001
2 (n = 13615)	Primary endpoint	2.32 (2.16–2.49)	< 0.001	1.925 (1.79–2.07)	< 0.001
	All-cause death	4.877 (4.23–5.62)	< 0.001	3.951 (3.42–4.56)	< 0.001
	SGLT2i-induced admission	1.676 (1.543–1.82)	< 0.001	1.41 (1.29–1.53)	< 0.001
≥ 3 (n = 13615)	Primary endpoint	4.151 (3.88–4.44)	< 0.001	3.04 (2.83–3.28)	< 0.001
	All-cause death	10.72 (9.36–12.29)	< 0.001	7.54 (6.53–8.71)	< 0.001
	SGLT2i-induced admission	2.52 (2.32–2.73)	< 0.001	1.87 (1.71–2.05)	< 0.001

* Multivariate Cox model were adjusted for age, sex, income, BMI, Charlson Comorbidity Index, hypertension, dyslipidemia, ischemic heart disease, atrial fibrillation, stroke, use of renin-angiotensin system blockade, beta-blockers, calcium channel blocker, antiplatelet agent, angiotensin receptor-neprilysin inhibitor, spironolactone, statins, loop diuretics, level of hemoglobin, serum glucose, creatinine, estimated GFR.

### Clinical outcomes in the propensity score-matched cohort

The group with primary endpoint occurrence exhibited significant differences in baseline characteristics compared to those without events. Therefore, separate cohorts were established for each variable that emerged as a significant factor in multivariable Cox analysis using propensity score-matching analysis. For all other factors, excluding the specific variable, covariates with a mean standardized difference of ≥ 0.1 were adjusted (S1 Fig in S1 File). In the propensity score-matching cohort, as in the original cohort, old age, lower BMI or body weight, decreased eGFR, anemia, use of loop diuretics and spironolactone emerged as independent predictors for the occurrence of primary composite events (Table 3).

### Sensitivity analysis of patients with cardiomyopathy or reduced ejection fraction

To determine whether the above results were consistently observed in specific HF patient groups, we conducted the same analysis in patients with congestive, dilated, and hypertrophic cardiomyopathy. Among the variables that were significant predictors of the primary endpoint in the entire population, old age (adjusted HR: 2.03, 95% CI: 1.55–2.65, *p* < 0.001), lower BMI (adjusted HR: 2.17, 95% CI: 1.03–4.55, *p* < 0.001), lower body weight (adjusted HR: 1.39, 95% CI: 1.04–1.86, *p* < 0.001), and use of loop diuretics (adjusted HR: 1.57, 95% CI: 1.16–2.13, *p* < 0.001) consistently emerged as independent risk factors in the sensitivity analysis using multivariable Cox analysis. Conversely, anemia (adjusted HR: 1.14, 95% CI: 0.85–1.52, *p* = 0.395), renal insufficiency (adjusted HR: 1.26, 95% CI: 0.97–1.63, *p* = 0.085), and use of spironolactone (adjusted HR: 0.89, 95% CI:0.69–1.16, ***p*** = 0.398) were not statistically significant.

## Discussion

We investigated the risk factors associated with discontinuation of SGLT2i due to adverse effects in patients with HF and diabetes. The adverse effects were defined as all-cause mortality or SGLT2i-induced admissions. We identified several risk factors for discontinuation due to SGLT2i-associated side effects, including age over 70 years, the use of diuretics (especially loop diuretics), low body weight or BMI, renal function impairment, and anemia. Age, use of diuretics, and body weight were also identified as consistent risk factors in the PS-matched cohort and in the subgroup with cardiomyopathy-related HF. To our knowledge, this study has the largest participant number in a real-world investigation of adverse effects related to SGLT2i and offers the advantage of conducting a sensitivity analysis in specific HF subgroups, including those with reduced ejection fraction. The identification of these risk factors and efforts to enhance patient adherence can contribute to the sustained prescription of beneficial SGLT2i, potentially improving outcomes in HF patients with diabetes.

In numerous studies highlighting the cardiovascular benefits of SGLT2i, the mean follow-up duration ranged from 2.4 to 4.2 years, with over 70% of participants continuing treatment for more than 2 years [3, 26, 27]. However, a meta-analysis encompassing 22 studies involving 123,000 participants revealed that less than 50% of the patients continued their treatment for a duration of 2 years in real-world clinical settings [13]. Such low adherence could compromise the efficacy of SGLT2i and hinder the realization of the beneficial effects of SGLT2i demonstrated in randomized controlled trial settings. Low adherence may be attributed to the occurrence of adverse effects such as genital infections, volume depletion, the onset of AKI, and the development of euglycemic DKA (EDKA) [28–30]. To prevent SGLT2i discontinuation, patients at higher risk should be informed at the time of prescription on minimizing adverse effects and how to address them. For instance, education on maintaining perineal hygiene is essential to prevent genital infections [31]. Moreover, to prevent side effects related to volume depletion, patients should be educated to maintain appropriate fluid and electrolyte intake. Patients should be advised to monitor their blood sugar levels or schedule an early outpatient visit if they experience symptoms such as polydipsia, nausea or vomiting, and abdominal pain, as these may indicate DKA. Our findings highlight the critical need for proactive education on the side effects of SGLT2i for diabetic patients with HF who possess multiple risk factors. Physicians should also closely monitor these high-risk patients for any adverse events.

Assessing the potential risk increase of DKA among SGLT2i users based on data from randomized control trials (RCTs) is challenging. In a 20-year retrospective cohort study from Denmark, the baseline incidence of DKA in type 2 diabetes patients was 1.34 per 1,000 person-years, with a decreasing annual incidence rate [32]. Most RCTs lacked the sample size needed to identify instances of DKA. Of the 16 RCTs mentioning DKA, only seven, involving 11,004 patients, documented at least one case [33]. EDKA is a form of diabetic ketoacidosis first recognized in the early 1970s [34]. With the increasing use of SGLT2 inhibitors, the incidence of EDKA has been rising [35]. This condition elevates glucagon levels through unclear mechanisms, which promotes ketone production [36]. SGLT2 inhibitors also induce negative fluid and sodium balance, exacerbating hypovolemic states and increasing cortisol, epinephrine, and glucagon levels, thereby increasing insulin resistance, ketogenesis, and lipolysis [35]. Clinically, EDKA resembles DKA with symptoms like nausea, vomiting, fatigue, and abdominal pain, but lacks significant hyperglycemia. This absence of marked hyperglycemia can mislead clinicians, delaying diagnosis and treatment and potentially worsening patient outcomes. Given the cardioprotective and renoprotective effects of SGLT2 inhibitors, their prescriptions have surged; however, the awareness and accurate diagnosis of EDKA remain insufficient. Our data indicate a high rate of unexplained hospitalizations and subsequent discontinuations of SGLT2i, suggesting possible inclusion of undiagnosed EDKA cases. These findings highlight the importance of vigilant monitoring of EDKA in high-risk patients to reduce discontinuation rates and improve cardiovascular outcomes.

In a recent randomized study, the use of empagliflozin in patients with HF taking furosemide resulted in increased urine volume and fluid clearance, and significant weight loss [37]. Notably, although the participants in this study had an average BMI and weight of 34 kg/m^2^ and 94kg, respectively, 22%–50% of those in the treatment arm of empagliflozin required a furosemide dose reduction [37]. In another similar study using dapagliflozin, the use of SGLT2i led to situations requiring discontinuation or dose reduction of furosemide (53.6% vs. 10.7%) [38]. In situations where the furosemide dose was not reduced, the dapagliflozin group experienced a higher incidence of major decline in renal function, defined as a decrease in kidney function by > 20% or a drop in eGFR to < 45% (28.6% vs. 0.0%). Our study, consistent with a recent small-scale study, found that high doses of loop diuretics increased the likelihood of discontinuing SGLT2i after discharge [28].

According to a recent study, upon discharge after hospitalization due to heart failure, almost all patients were prescribed more than five medications, and more than half were prescribed over ten medications [39]. Although the compliance of diabetic patients with SGLT2i in real clinical practice is not high [13, 28], polypharmacy itself may be contribute to the discontinuation of SGLT2i. Therefore, it is necessary to develop strategies to reduce the negative impact of polypharmacy in patients with HF.

This study has several limitations. First, in Korea, SGLT2i can be claimed for insurance reimbursement as for diabetes treatment but not for HF. Consequently, we could not investigate the risk factors SGLT2i discontinuation in patients with HF without diabetes. Nevertheless, considering the low proportion of DKA in the primary endpoints and consistent efficacy of SGLT2i in both non-diabetic and diabetic patients in previous studies [3, 5, 6, 10], we hypothesize that our findings might be applicable to non-diabetic patients with HF. However, further studies are needed to confirm this hypothesis, as the mechanism of action of SGLT2 inhibitors may differ between diabetic and non-diabetic patients. Second, we only included patients who used dapagliflozin and empagliflozin, potentially introducing selection bias. These drugs were selected as they are widely used for HF. Third, our study lacked data on laboratory parameters such as HbA1c and the history of using other diabetes-related medications. As mentioned earlier, this limitation arises because our study primarily focuses on diabetic patients with HF and not solely on diabetes. Additionally, we lacked laboratory findings such as NT-proBNP that are challenging to extract from health screening data and echocardiographic parameters such as diastolic dysfunction. This is an inherent limitation of claim data analysis. Fourth, we did not perform external validation with other databases. However, given that this is a nationwide big-data study and similar results have been observed in smaller previous study [28], the robustness of our findings remains credible. Lastly, this study was performed in a Korean population, and further research is required to determine if similar results emerge in other ethnic groups.

## Conclusion

We identified several risk factors that deter the continuous use of SGLT2i in diabetic patients with HF. These risk factors include old age, low weight or low BMI, anemia, renal insufficiency, and the use of diuretics. For patients with multiple risk factors, close monitoring of side effects should a priority when prescribing SGLT2i.

## Supporting information

S1 File (DOCX)

S1 Graphical abstract (TIF)

## References

[1] Heerspink HJL, Stefansson BV, Correa-Rotter R, Chertow GM, Greene T, Hou FF, et al. Dapagliflozin in Patients with Chronic Kidney Disease. N Engl J Med. 2020;383(15):1436–46. doi: 10.1056/NEJMoa2024816 . 32970396

[2] Wiviott SD, Raz I, Bonaca MP, Mosenzon O, Kato ET, Cahn A, et al. Dapagliflozin and Cardiovascular Outcomes in Type 2 Diabetes. N Engl J Med. 2019;380(4):347–57. doi: 10.1056/NEJMoa1812389 . 30415602

[3] McMurray JJV, Solomon SD, Inzucchi SE, Kober L, Kosiborod MN, Martinez FA, et al. Dapagliflozin in Patients with Heart Failure and Reduced Ejection Fraction. N Engl J Med. 2019;381(21):1995–2008. doi: 10.1056/NEJMoa1911303 . 31535829

[4] Zinman B, Wanner C, Lachin JM, Fitchett D, Bluhmki E, Hantel S, et al. Empagliflozin, Cardiovascular Outcomes, and Mortality in Type 2 Diabetes. N Engl J Med. 2015;373(22):2117–28. doi: 10.1056/NEJMoa1504720 . 26378978

[5] Solomon SD, McMurray JJV, Claggett B, de Boer RA, DeMets D, Hernandez AF, et al. Dapagliflozin in Heart Failure with Mildly Reduced or Preserved Ejection Fraction. N Engl J Med. 2022;387(12):1089–98. doi: 10.1056/NEJMoa2206286 . 36027570

[6] Anker SD, Butler J, Filippatos G, Ferreira JP, Bocchi E, Bohm M, et al. Empagliflozin in Heart Failure with a Preserved Ejection Fraction. N Engl J Med. 2021;385(16):1451–61. doi: 10.1056/NEJMoa2107038 . 34449189

[7] Lopaschuk GD, Verma S. Mechanisms of Cardiovascular Benefits of Sodium Glucose Co-Transporter 2 (SGLT2) Inhibitors: A State-of-the-Art Review. JACC Basic Transl Sci. 2020;5(6):632–44. doi: 10.1016/j.jacbts.2020.02.004 . 32613148 PMC7315190

[8] Mascolo A, Di Napoli R, Balzano N, Cappetta D, Urbanek K, De Angelis A, et al. Safety profile of sodium glucose co-transporter 2 (SGLT2) inhibitors: A brief summary. Front Cardiovasc Med. 2022;9:1010693. doi: 10.3389/fcvm.2022.1010693 . 36211584 PMC9532622

[9] Mukai J, Kanno S, Kubota R. A literature review and meta-analysis of safety profiles of SGLT2 inhibitors in Japanese patients with diabetes mellitus. Sci Rep. 2021;11(1):13472. doi: 10.1038/s41598-021-92925-2 . 34188120 PMC8241876

[10] Packer M, Anker SD, Butler J, Filippatos G, Pocock SJ, Carson P, et al. Cardiovascular and Renal Outcomes with Empagliflozin in Heart Failure. N Engl J Med. 2020;383(15):1413–24. doi: 10.1056/NEJMoa2022190 . 32865377

[11] Huang CY, Lee JK. Sodium-glucose co-transporter-2 inhibitors and major adverse limb events: A trial-level meta-analysis including 51 713 individuals. Diabetes Obes Metab. 2020;22(12):2348–55. doi: 10.1111/dom.14159 . 32744411

[12] Talha KM, Anker SD, Butler J. SGLT-2 Inhibitors in Heart Failure: A Review of Current Evidence. Int J Heart Fail. 2023;5(2):82–90. doi: 10.36628/ijhf.2022.0030 . 37180562 PMC10172076

[13] Ofori-Asenso R, Sahle BW, Chin KL, Mazidi M, Ademi Z, De Bruin ML, et al. Poor adherence and persistence to sodium glucose co-transporter 2 inhibitors in real-world settings: Evidence from a systematic review and meta-analysis. Diabetes Metab Res Rev. 2021;37(1):e3350. doi: 10.1002/dmrr.3350 . 32447808

[14] McDonagh TA, Metra M, Adamo M, Gardner RS, Baumbach A, Bohm M, et al. 2021 ESC Guidelines for the diagnosis and treatment of acute and chronic heart failure. Eur Heart J. 2021;42(36):3599–726. doi: 10.1093/eurheartj/ehab368 . 34447992

[15] Cosentino F, Grant PJ, Aboyans V, Bailey CJ, Ceriello A, Delgado V, et al. 2019 ESC Guidelines on diabetes, pre-diabetes, and cardiovascular diseases developed in collaboration with the EASD. Eur Heart J. 2020;41(2):255–323. doi: 10.1093/eurheartj/ehz486 . 31497854

[16] Lee J, Lee JS, Park SH, Shin SA, Kim K. Cohort Profile: The National Health Insurance Service-National Sample Cohort (NHIS-NSC), South Korea. Int J Epidemiol. 2017;46(2):e15. doi: 10.1093/ije/dyv319 . 26822938

[17] Kim TH, Yang PS, Yu HT, Jang E, Shin H, Kim HY, et al. Effect of hypertension duration and blood pressure level on ischaemic stroke risk in atrial fibrillation: nationwide data covering the entire Korean population. Eur Heart J. 2019;40(10):809–19. doi: 10.1093/eurheartj/ehy877 . 30608537

[18] Kim K, Park SM, Lee K. Weight gain after smoking cessation does not modify its protective effect on myocardial infarction and stroke: evidence from a cohort study of men. Eur Heart J. 2018;39(17):1523–31. doi: 10.1093/eurheartj/ehx761 . 29324990 PMC5930246

[19] Choi YJ, Kim SH, Kang SH, Yoon CH, Lee HY, Youn TJ, et al. Reconsidering the cut-off diastolic blood pressure for predicting cardiovascular events: a nationwide population-based study from Korea. Eur Heart J. 2019;40(9):724–31. doi: 10.1093/eurheartj/ehy801 . 30535368

[20] Choi YJ, Choi EK, Han KD, Jung JH, Park J, Lee E, et al. Temporal trends of the prevalence and incidence of atrial fibrillation and stroke among Asian patients with hypertrophic cardiomyopathy: A nationwide population-based study. Int J Cardiol. 2018;273:130–5. doi: 10.1016/j.ijcard.2018.08.038 . 30150122

[21] Sundararajan V, Henderson T, Perry C, Muggivan A, Quan H, Ghali WA. New ICD-10 version of the Charlson comorbidity index predicted in-hospital mortality. J Clin Epidemiol. 2004;57(12):1288–94. doi: 10.1016/j.jclinepi.2004.03.012 . 15617955

[22] Charlson ME, Pompei P, Ales KL, MacKenzie CR. A new method of classifying prognostic comorbidity in longitudinal studies: development and validation. J Chronic Dis. 1987;40(5):373–83. doi: 10.1016/0021-9681(87)90171-8 . 3558716

[23] Cappellini MD, Motta I. Anemia in Clinical Practice-Definition and Classification: Does Hemoglobin Change With Aging? Semin Hematol. 2015;52(4):261–9. doi: 10.1053/j.seminhematol.2015.07.006 . 26404438

[24] Skali H, Uno H, Levey AS, Inker LA, Pfeffer MA, Solomon SD. Prognostic assessment of estimated glomerular filtration rate by the new Chronic Kidney Disease Epidemiology Collaboration equation in comparison with the Modification of Diet in Renal Disease Study equation. Am Heart J. 2011;162(3):548–54. doi: 10.1016/j.ahj.2011.06.006 . 21884875

[25] Park JB. Using Big Data to Understand Rare Diseases. Int J Heart Fail. 2021;3(3):194–6. doi: 10.36628/ijhf.2021.0026 . 36262643 PMC9536653

[26] Neal B, Perkovic V, Mahaffey KW, de Zeeuw D, Fulcher G, Erondu N, et al. Canagliflozin and Cardiovascular and Renal Events in Type 2 Diabetes. N Engl J Med. 2017;377(7):644–57. doi: 10.1056/NEJMoa1611925 . 28605608

[27] Bode B, Stenlof K, Harris S, Sullivan D, Fung A, Usiskin K, et al. Long-term efficacy and safety of canagliflozin over 104 weeks in patients aged 55–80 years with type 2 diabetes. Diabetes Obes Metab. 2015;17(3):294–303. doi: 10.1111/dom.12428 . 25495720

[28] Nakagaito M, Imamura T, Ushijima R, Nakamura M, Kinugawa K. Predictors and Outcomes of SGLT2 Inhibitor Discontinuation in a Real-World Population after Hospitalization for Heart Failure. Biomedicines. 2023;11(3). doi: 10.3390/biomedicines11030876 . 36979855 PMC10046005

[29] Liu J, Li L, Li S, Jia P, Deng K, Chen W, et al. Effects of SGLT2 inhibitors on UTIs and genital infections in type 2 diabetes mellitus: a systematic review and meta-analysis. Sci Rep. 2017;7(1):2824. doi: 10.1038/s41598-017-02733-w . 28588220 PMC5460243

[30] Goldenberg RM, Berard LD, Cheng AYY, Gilbert JD, Verma S, Woo VC, et al. SGLT2 Inhibitor-associated Diabetic Ketoacidosis: Clinical Review and Recommendations for Prevention and Diagnosis. Clin Ther. 2016;38(12):2654–64.e1. doi: 10.1016/j.clinthera.2016.11.002 . 28003053

[31] Kalra S, Baruah MP, Sahay R. Medication counselling with sodium glucose transporter 2 inhibitor therapy. Indian J Endocrinol Metab. 2014;18(5):597–9. doi: 10.4103/2230-8210.139206 . 25285273 PMC4171879

[32] Jensen ML, Persson F, Andersen GS, Ridderstrale M, Nolan JJ, Carstensen B, et al. Incidence of Ketoacidosis in the Danish Type 2 Diabetes Population Before and After Introduction of Sodium-Glucose Cotransporter 2 Inhibitors-A Nationwide, Retrospective Cohort Study, 1995–2014. Diabetes Care. 2017;40(5):e57–e8. doi: 10.2337/dc16-2793 . 28283564

[33] Donnan JR, Grandy CA, Chibrikov E, Marra CA, Aubrey-Bassler K, Johnston K, et al. Comparative safety of the sodium glucose co-transporter 2 (SGLT2) inhibitors: a systematic review and meta-analysis. BMJ Open. 2019;9(1):e022577. doi: 10.1136/bmjopen-2018-022577 . 30813108 PMC6361337

[34] Munro JF, Campbell IW, McCuish AC, Duncan LJ. Euglycaemic diabetic ketoacidosis. Br Med J. 1973;2(5866):578–80. doi: 10.1136/bmj.2.5866.578 . 4197425 PMC1592207

[35] Chow E, Clement S, Garg R. Euglycemic diabetic ketoacidosis in the era of SGLT-2 inhibitors. BMJ Open Diabetes Res Care. 2023;11(5). doi: 10.1136/bmjdrc-2023-003666 . 37797963 PMC10551972

[36] Keller U, Schnell H, Sonnenberg GE, Gerber PP, Stauffacher W. Role of glucagon in enhancing ketone body production in ketotic diabetic man. Diabetes. 1983;32(5):387–91. doi: 10.2337/diab.32.5.387 . 6132846

[37] Mordi NA, Mordi IR, Singh JS, McCrimmon RJ, Struthers AD, Lang CC. Renal and Cardiovascular Effects of SGLT2 Inhibition in Combination With Loop Diuretics in Patients With Type 2 Diabetes and Chronic Heart Failure: The RECEDE-CHF Trial. Circulation. 2020;142(18):1713–24. doi: 10.1161/CIRCULATIONAHA.120.048739 . 32865004 PMC7594536

[38] Singh JSS, Mordi IR, Vickneson K, Fathi A, Donnan PT, Mohan M, et al. Dapagliflozin Versus Placebo on Left Ventricular Remodeling in Patients With Diabetes and Heart Failure: The REFORM Trial. Diabetes Care. 2020;43(6):1356–9. doi: 10.2337/dc19-2187 . 32245746 PMC7245350

[39] Unlu O, Levitan EB, Reshetnyak E, Kneifati-Hayek J, Diaz I, Archambault A, et al. Polypharmacy in Older Adults Hospitalized for Heart Failure. Circ Heart Fail. 2020;13(11):e006977. doi: 10.1161/CIRCHEARTFAILURE.120.006977 . 33045844 PMC8819498

