# Supplementary material for: Risk factors associated with SGLT2 inhibitor discontinuation in diabetic patients with heart failure
Source: PLoS One. 2024 Nov 25;19(11):e0314305. doi: 10.1371/journal.pone.0314305 (PMC11588271; doi:10.1371/journal.pone.0314305)
Supplement: S1 File — (DOCX) [file pone.0314305.s001.docx]

**Risk Factors Associated with SGLT2 Inhibitor Discontinuation in Diabetic Patients with Heart Failure: A Nationwide Cohort Study**

Minkwan Kim^1†^, Seok-Jae Heo^2†^­, Moon-Hyun Kim^1^, Je-Wook Park^1^, SungA Bae^1^, Ji Woong Roh^1^, Oh-Hyun Lee^1^, Yongcheol Kim^1^, Eui Im^1^, In Hyun Jung^1^*, Deok-Kyu Cho^1^

^1^Division of Cardiology, Department of Internal Medicine, Yongin Severance Hospital, Yonsei University College of Medicine, Yongin, Gyeonggi-do, Republic of Korea

^2^Division of Biostatistics, Department of Biomedical Systems Informatics, Yonsei University College of Medicine, Seoul, Republic of Korea

^†^ These two authors contributed equally to this work.

* Corresponding author: In Hyun Jung

**I. Supplementary Tables**

**Supplementary Table 1. Cox Regression Analysis of Original and Propensity Score-Matched Cohorts of SGLT2i-Treated Diabetic Patients with HF**

|  |  | **Univariable Cox** | | **Multivariable Cox*** | | **Propensity score-matched cohort*** | |
| --- | --- | --- | --- | --- | --- | --- | --- |
| **Variables** |  | **HR (95% CI)** | ***p*** | **HR (95% CI)** | ***p*** | **HR (95% CI)** | ***p*-value** |
| Age ≥ 70 years | Primary endpoint | 2.19 (2.10–2.29) | < 0.001 | 1.66 (1.59–1.75) | < 0.001 | 1.45 (1.36–1.54) | < 0.001 |
|  | All-cause death | 3.94 (3.65–4.27) | < 0.001 | 2.764 (2.54–3.01) | < 0.001 | 2.74 (2.32–3.23) | < 0.001 |
|  | SGLT2i-induced admission | 1.57 (1.48–1.66) | < 0.001 | 1.25 (1.18–1.33) | < 0.001 | 1.27 (1.19–1.36) | < 0.001 |
| BMI < 18.5 kg/m^2^ | Primary endpoint | 3.01 (2.57–3.52) | < 0.001 | 1.82 (1.54–2.13) | < 0.001 | 1.78 (1.29–2.45) | < 0.001 |
|  | All-cause death | 4.97 (4.05–6.08) | < 0.001 | 2.24 (1.81–2.77) | < 0.001 | 3.14 (1.72–5.74) | < 0.001 |
|  | SGLT2i-induced admission | 1.88 (1.46–2.41) | < 0.001 | 1.41 (1.10–1.82) | 0.008 | 1.30 (0.89–1.90) | 0.179 |
| Body weight < 60kg | Primary endpoint | 1.54 (1.47–1.61) | < 0.001 | 1.25 (1.18–1.31) | < 0.001 | 1.17 (1.09–1.26) | < 0.001 |
|  | All-cause death | 1.90 (1.77–2.04) | < 0.001 | 1.55 (1.43–1.68) | < 0.001 | 1.73 (1.46–2.04) | < 0.001 |
|  | SGLT2i-induced admission | 1.33 (1.25–1.41) | < 0.001 | 1.08 (1.01–1.16) | 0.022 | 1.07 (0.99–1.16) | 0.086 |
| Fasting glucose ≥ 126mg/dL | Primary endpoint | 0.93 (0.90–0.98) | 0.002 | 1.04 (1.00–1.09) | 0.084 | 1.00 (0.94–1.05) | 0.865 |
|  | All-cause death | 0.98 (0.91–1.05) | 0.541 | 1.15 (1.07–1.23) | < 0.001 | 1.07 (0.94–1.21) | 0.314 |
|  | SGLT2i-induced admission | 0.91 (0.86–0.96) | < 0.001 | 0.98 (0.93–1.04) | 0.473 | 0.98 (0.92–1.04) | 0.489 |
| Hemoglobin < 13 (male) | Primary endpoint | 2.09 (1.99–2.19) | < 0.001 | 1.41 (1.34–1.48) | < 0.001 | 1.32 (1.22–1.43) | < 0.001 |
| or < 12 (female) g/dL |  | 2.81 (2.61–3.02) | < 0.001 | 1.616 (1.50–1.75) | < 0.001 | 1.78 (1.49–2.11) | < 0.001 |
|  | All-cause death | 1.67 (1.56–1.79) | < 0.001 | 1.25 (1.16–1.34) | < 0.001 | 1.21 (1.10–1.32) | < 0.001 |
|  | SGLT2i-induced admission | 1.69 (1.62–1.77) | < 0.001 | 1.21 (1.15–1.27) | < 0.001 | 1.17 (1.09–1.25) | < 0.001 |
| Estimated GFR < 60mL/min/1.73m^2^ | Primary endpoint | 2.35 (2.19–2.51) | < 0.001 | 1.28 (1.19–1.39) | < 0.001 | 1.29 (1.12–1.49) | < 0.001 |
|  | All-cause death | 1.37 (1.29–1.443) | < 0.001 | 1.17 (1.10–1.25) | < 0.001 | 1.14 (1.06–1.22) | < 0.001 |
|  | SGLT2i-induced admission | 1.98 (1.90–2.08) | < 0.001 | 1.38 (1.30–1.46) | < 0.001 | 1.36 (1.25–1.47) | < 0.001 |
| Use of loop diuretics | Primary endpoint | 2.53 (2.36–2.71) | < 0.001 | 1.57 (1.45–1.71) | < 0.001 | 1.45 (1.22–1.74) | < 0.001 |
|  | All-cause death | 1.68 (1.59–1.79) | < 0.001 | 1.26 (1.17–1.35) | < 0.001 | 1.33 (1.21–1.46) | < 0.001 |
|  | SGLT2i-induced admission | 1.81 (1.72–1.91) | < 0.001 | 1.20 (1.12–1.28) | < 0.001 | 1.20 (1.10–1.31) | < 0.001 |
| Use of MRA | Primary endpoint | 2.12 (1.95–2.30) | < 0.001 | 1.19 (1.08–1.32) | < 0.001 | 1.24 (1.03–1.50) | 0.027 |
|  | All-cause death | 1.63 (1.52–1.75) | < 0.001 | 1.22 (1.12–1.33) | < 0.001 | 1.20 (1.09–1.32) | < 0.001 |
|  | SGLT2i-induced admission | 2.19 (2.10–2.29) | < 0.001 | 1.66 (1.59–1.75) | < 0.001 | 1.45 (1.36–1.54) | < 0.001 |

* Multivariate Cox and propensity score-matching model were adjusted for age, sex, income, BMI, Charlson Comorbidity Index, hypertension, dyslipidemia, ischemic heart disease, atrial fibrillation, stroke, use of renin-angiotensin system blockade, beta-blockers, calcium channel blocker, antiplatelet agent, angiotensin receptor-neprilysin inhibitor, MRA, statins, loop diuretics, level of hemoglobin, serum glucose, creatinine, estimated GFR. In each statistical test, independent variables were excluded from the adjustment. HR, hazard ratio

**Supplementary Table 2. Definitions of covariates.**

| **Diagnosis** | **ICD-10-CM code and definition** | **Diagnostic definition** |
| --- | --- | --- |
| **Hypertension** | I10-I13, I15; and minimum 1 prescription of anti-hypertensive drug (thiazide, loop diuretics, aldosterone antagonist, alpha-/beta-blocker, calcium channel blocker, renin-angiotensin system blocker). | Admission≥1 or outpatient department≥2 |
| **Dyslipidemia** | E78, and minimum 1 prescription of lipid-lowering medication (statin, ezetimibe, fenofibrate) | Admission≥1 or outpatient department≥2 |
| **Ischemic heart disease** | I20, I23-25; and procedure code of M6561-6567 | Admission ≥ 1 or outpatient clinic ≥ 2 |
| **Heart failure** | I50 | Admission≥1 or outpatient clinic ≥ 2 |
| **Atrial fibrillation** | I48 | Admission≥1 or outpatient clinic ≥ 2 |
| **Previous stroke** | I63, I64 | Admission≥1 or outpatient clinic ≥ 2 |
| **Chronic kidney disease** | N00-007, N11, I12, N18-19, Q61 | Admission≥1 or outpatient clinic ≥ 2 |

**II. Supplementary Figures**

**Supplementary Figure 1. Mean standardised differences after propensity score-matching analyses
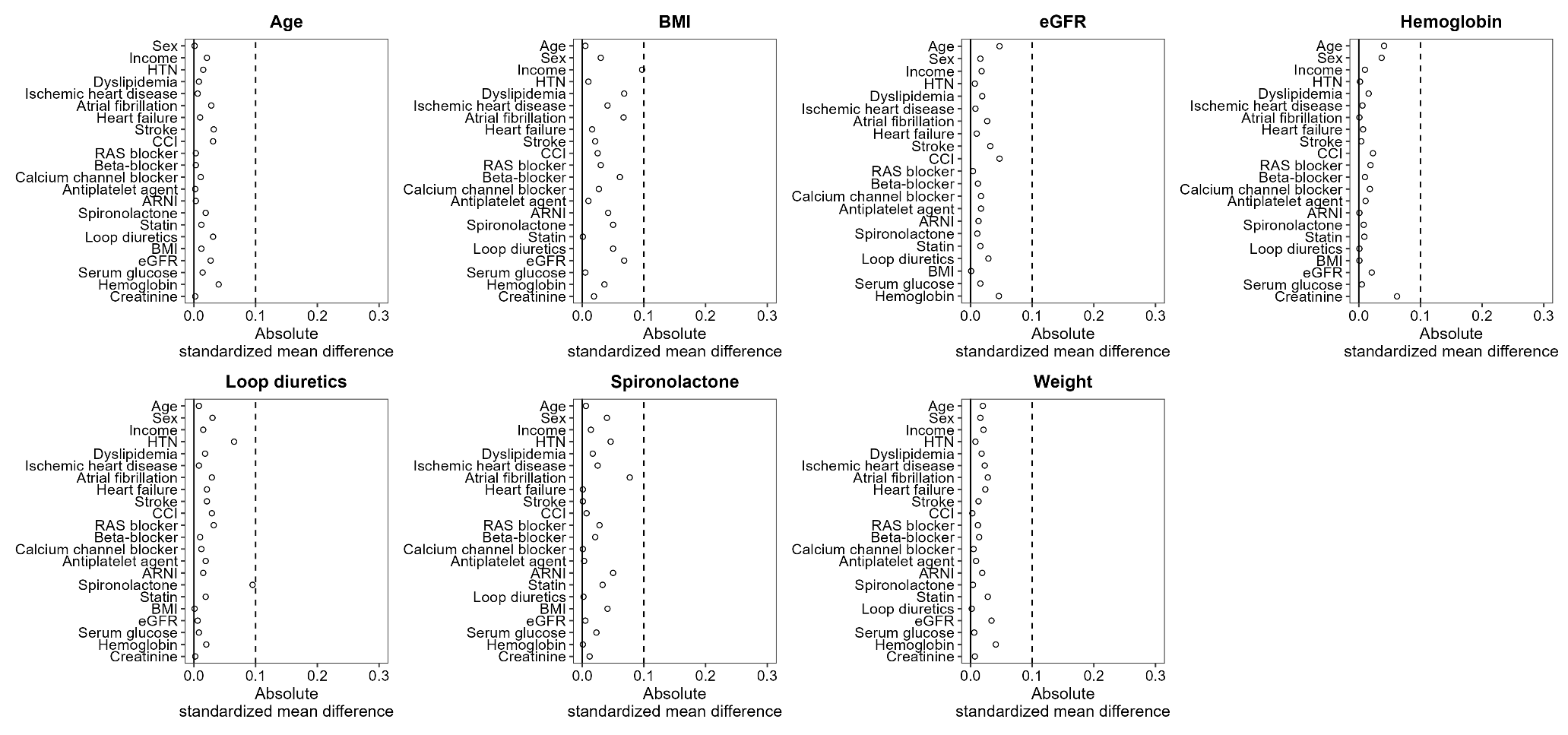
**
