# Supplementary figures and images for: Risk factors associated with SGLT2 inhibitor discontinuation in diabetic patients with heart failure
Source: PLoS One. 2024 Nov 25;19(11):e0314305. doi: 10.1371/journal.pone.0314305 (PMC11588271; doi:10.1371/journal.pone.0314305)

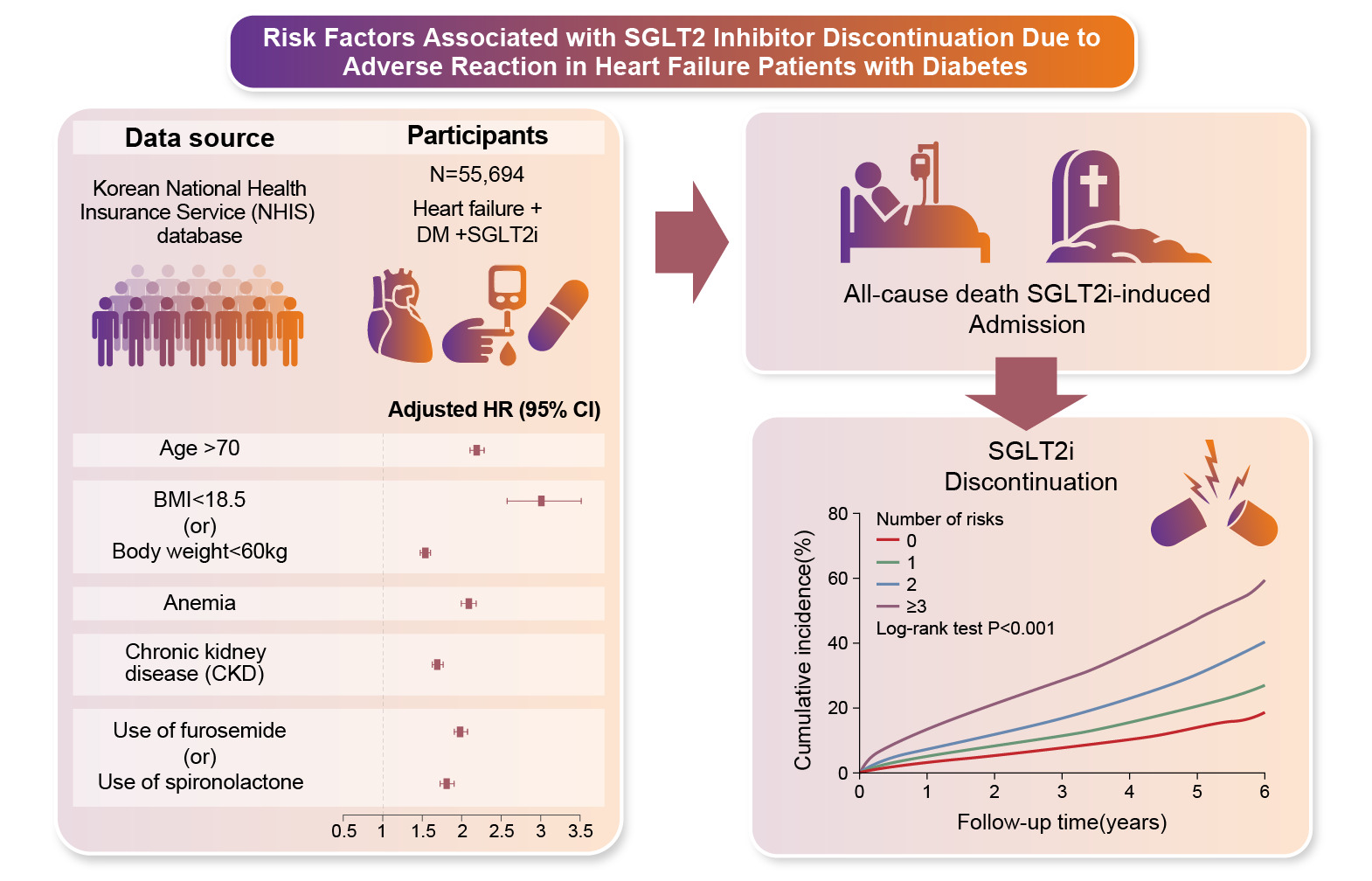

Supplement: S1 Graphical abstract — (TIF) [file pone.0314305.s002.tif]
